# Near-Infrared-Emissive AIE Bioconjugates: Recent Advances and Perspectives

**DOI:** 10.3390/molecules27123914

**Published:** 2022-06-18

**Authors:** Wenshuai Luo, Yonghong Tan, Yixiong Gui, Dingyuan Yan, Dong Wang, Ben Zhong Tang

**Affiliations:** 1 Center for AIE Research, Shenzhen Key Laboratory of Polymer Science and Technology, Guangdong Research Center for Interfacial Engineering of Functional Materials, College of Materials Science and Engineering, Shenzhen University, Shenzhen 518060, China; 2070343008@email.szu.edu.cn (W.L.); 2110343013@email.szu.edu.cn (Y.T.); 2070343099@email.szu.edu.cn (Y.G.); 2 School of Science and Engineering, Shenzhen Institute of Aggregate Science and Technology, The Chinese University of Hong Kong, Shenzhen 518172, China

**Keywords:** aggregation-induced emission, NIR emission, bioconjugates, biomedical applications

## Abstract

Near-infrared (NIR) fluorescence materials have exhibited formidable power in the field of biomedicine, benefiting from their merits of low autofluorescence background, reduced photon scattering, and deeper penetration depth. Fluorophores possessing planar conformation may confront the shortcomings of aggregation-caused quenching effects at the aggregate level. Fortunately, the concept of aggregation-induced emission (AIE) thoroughly reverses this dilemma. AIE bioconjugates referring to the combination of luminogens showing an AIE nature with biomolecules possessing specific functionalities are generated via the covalent conjugation between AIEgens and functional biological species, covering carbohydrates, peptides, proteins, DNA, and so on. This perfect integration breeds unique superiorities containing high brightness, good water solubility, versatile functionalities, and prominent biosafety. In this review, we summarize the recent progresses of NIR-emissive AIE bioconjugates focusing on their design principles and biomedical applications. Furthermore, a brief prospect of the challenges and opportunities of AIE bioconjugates for a wide range of biomedical applications is presented.

## 1. Introduction

Research on biological events is of great scientific importance and has been intensively investigated aiming to detect the progression of diseases and implement interventions in time [1,2,3]. Although multifarious imaging tools for the detection of pathological changes, including electron-based microscopy, positron emission-based computed tomography (CT), magnetic resonance-based imaging (MRI), etc., have been developed, the limitations of hazardous ionizing radiation, time- and money-consuming, complex operational process, and low spatial and temporal resolution seriously affect their widespread applications in daily usage [4,5,6,7]. In contrast, the advent of fluorescence technology provides a powerful alternative tool to visualize biological events, given its distinctive advantages in simplicity, high sensitivity, high spatial and temporal resolution, and non-invasive operation process [8,9,10].

Under the circumstances, plenty of fluorescent probes, such as fluorescent proteins, organic dyes, and inorganic nanoparticles (NPs), have been elaborately manipulated and exhibited tremendous utility in the field of bioimaging [11,12,13]. Among the above-mentioned fluorescent materials, inorganic NPs such as quantum dots or up-conversion NPs bring about inevitable biocompatibility concerns given their undefined purity and are hard to degrade, which severely restrains the clinical translations compared to organic small molecules [14]. In particular, fluorescence imaging in the broadly defined near-infrared (NIR) window which encompasses NIR-I (700–900 nm) and NIR-II (1000–1700 nm) regions possesses numerous advantages, such as deeper penetration depth, reduced photon scattering, and minimized autofluorescence background, allowing for an overall superior imaging performance [15,16,17,18]. However, most of the NIR-emissive fluorescent probes are prone to form aggregates in the physiological environment as the extension of the π-conjugated backbone. In this case, conventional chromophores that are composed of planar fragments may suffer from aggregation-caused quenching (ACQ) effects due to the extensive prevalence of intermolecular π−π interactions, thus hampering their applications both in sensitivity and accuracy [19].

Fortunately, luminogens displaying aggregation-induced emission (AIE) characteristics that are thoroughly the opposite to ACQ effects provide a good solution for bioimaging even in an aggregated state [20,21]. According to the mechanism of restricted intramolecular motion (RIM), the nonradiative energy decay pathway of AIE luminogens (AIEgens) is suppressed upon the propeller-like architecture coming together (forming aggregates), therefore boosting the fluorescence intensity [22,23,24]. The inherent emitting attributes of AIEgens endow their unique behaviors, such as large Stokes shift, superior photostability, and reliable output signal at high detection concentrations, compared to traditional fluorescent probes [25,26]. In addition, the AIE property permits AIEgens to show a fluorescence turn-on feature when spontaneously aggregated in a hydrophilic environment or couple to an analyte, providing high sensitivity and a good signal-to-noise ratio (SNR) [27,28,29,30,31].

AIE bioconjugates are species that are formulated by chemical strategies in which biomolecules such as carbohydrates, peptides, enzymes, proteins, DNA, and other biological species are directly binding to AIEgens through stable covalent bonds, or attached on the surface of AIEgens NPs [32,33,34,35,36,37]. The resultant nanocomposites can concurrently share the integrated merits of imaging and/or therapy functions originating from AIEgens and targeting capacity deriving from biological elements [38,39].

Considering that AIEgens are weak or non-emissive in a soluble state, strategies containing ionization, PEGylation, etc., are adopted for ensuring their water solubility or hydrophilicity, thus reducing background noise, and improving sensitivity [40,41]. However, the selectivity and specificity are difficult to guarantee. The conjugation of AIEgens with hydrophilic functional carbohydrates, peptides, or other species on the one hand provides an alternative to endow the resultant bioconjugates with a specific affinity [42]. On the other hand, the hydrophily of AIEgens may obviously increase after coupling with hydrophilic counterparts, permitting a low-emission background and a high sensitivity for subsequent bioassays [25,43]. In addition, the biocompatibility of AIE bioconjugates is excellent owing to the inherently biological components. Hence, AIE bioconjugates undoubtedly offer a powerful tool that not only opens new avenues for the construction of a host of bioprobes, but also bears versatile additional functionalities [44].

**Figure 1:**
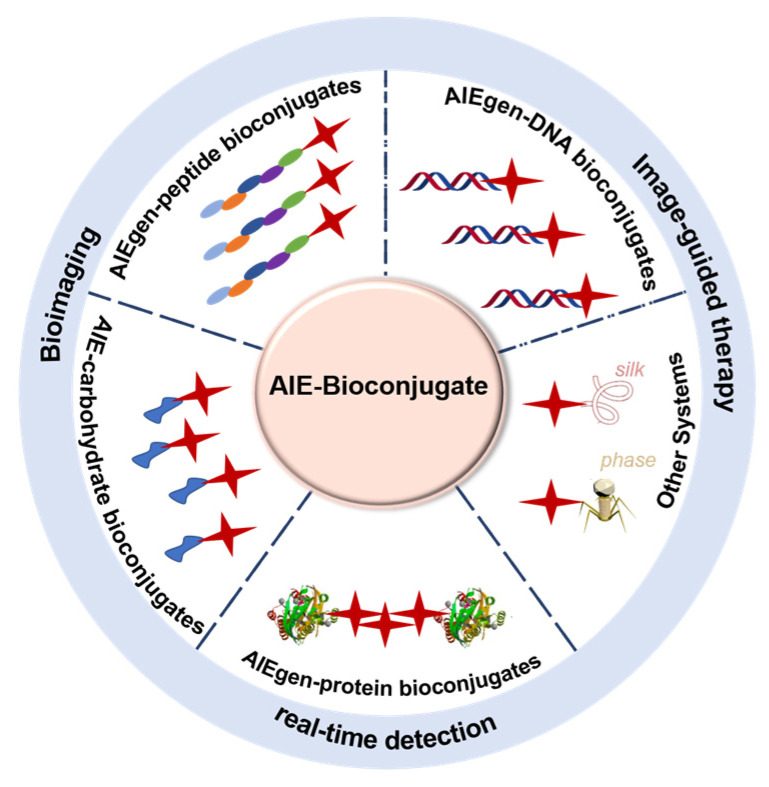
Schematic diagram of the representative bioconjugate coupling with NIR-emissive AIEgens.

Inspired by the above, we highlight the recent advancements of AIE bioconjugates that emit NIR fluorescence and their biomedical applications (Figure 1). Furthermore, the challenges and prospects in this field are shortly discussed. Representative examples classified based on different conjugated biomolecules involving AIE-carbohydrate bioconjugates, AIE-peptide bioconjugates, AIE-protein bioconjugates, and others will be discussed in detail.

## 2. AIE-Carbohydrate Bioconjugates

**Figure 2:**
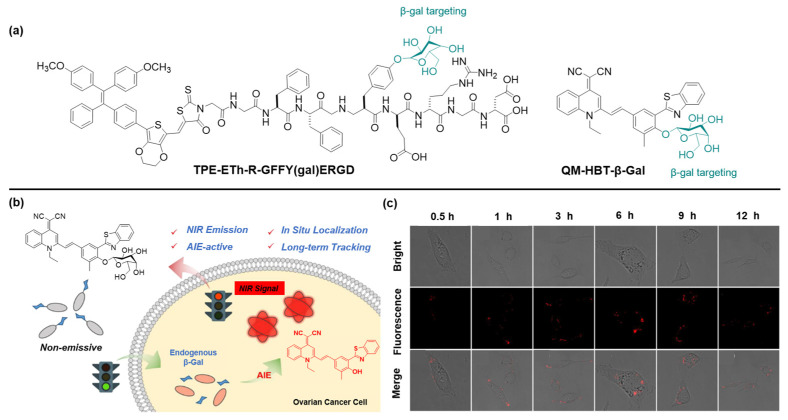
(**a**) Structures of β-gal-targeting probes. (**b**) Schematic diagram of QM-HBT-β-gal towards β-gal. (**c**) Long-term tracking capability of QM-HBT-β-gal. Copyright 2019, Frontier Media.

By selectively identifying and removing senescent cancer cells, it is possible to extend life and improve the effectiveness of cancer treatment [45]. β-Galactosidase (β-gal), an important enzymatic reporter, plays a key role in a wide range of biological processes [46]. It is widely accepted that β-gal is tightly relevant to primary tumorigenesis and metastasis [47]. Thus, the development of accurate and rapid methods for monitoring the activity of β-gal is essential for early cancer diagnosis and biological research. Recently, Gao et al., elaborately designed an enzyme-instructed self-assembly (EISA) strategy to specifically target and ablate senescent HeLa (s-HeLa) cells [48]. Taking the feature of overexpressed β-gal in s-HeLa, probe TPE-ETh-R-GFFY(gal)ERGD can effectively accumulate and switch on its fluorescence after recognition with β-gal (Figure 2a). Moreover, the probe can generate reactive oxygen species (ROS) and then kill senescent cells under the light illumination.

Fu et al. developed an activable β-gal probe, QM-HBT-β-gal, that is composed of an AIE-active core quinoline-malononitrile (QM) with a hydrophobic 2-(2-hydroxyphenyl) benzothiazole (HBT) moiety for prolonging the wavelength into the NIR region, and a hydrophilic β-gal responsive moiety (Figure 2c) [49]. As expected, the monomolecular disperse state in the aqueous environment endows the probe with weak fluorescent emission. When hydrolyzed by β-gal, hydrophobic QM-HBT-OH can be released and aggregated with a remarkable light-up fluorescent signal, which could be well-retained in the reaction site and emit strong fluorescence for long-term tracking of endogenous β-gal activity. To verify that the AIE-active strategy could achieve on-site sensing and long-term tracking of endogenous β-gal in living cells, SKOV-3 cells were selected to study the confocal laser scanning microscopy (CLSM) images after incubatation with QM-HBT-β-gal. As shown in Figure 2c, its fluorescence intensity gradually boosted and attained the maximum level 3 h post-incubation, showcasing the aggregation of QM-HBT-OH. As the incubation time further extended to 12 h, the intracellular fluorescence intensity weakened a little. Altogether, the results of the cell experiment revealed that the probe QM-HBT-β-gal can overcome intracellular diffusion and attain high-fidelity enzyme information, permitting real-time detection of β-gal in SKOV-3 cells. Considering the high performance of QM-HBT-β-gal, the AIE-active strategy paves a new pathway for in situ and long-term tracking of enzyme activity in preclinical applications.

## 3. AIEgen-Peptide Bioconjugates

### 3.1. Activatable AIEgen-Peptide Bioconjugates

In imaging-guided therapy, activity-based sensing offers unique advantages such as low systematic interventions and excellent selectivity [25,50]. Typically, responsive probes do not generate a fluorescent signal until they are lit-up by targeting goals, thus showing a high SNR [51]. In the organism, enzymes are widely distributed and participate in many physical activities. The occurrence and development of disease is closely related to the activity or the expression level of enzymes [52,53,54]. For instance, matrix metalloproteinases (MMPs) serve a vital role in the progression of disorders, such as tissue repair and reconstruction, arthritis, and cancer [55]. Among diverse activity-based bioprobes, peptide-modified AIE bioprobes combine obvious advantages, such as excellent specificity, better sensitivity, inherent biological activity, and abundant diversity [41]. Owing to the introduction of peptides, the hydrophilicity of AIE molecules could be significantly improved. Once the peptide fragment is specifically cleaved by enzymes, the fluorescence intensity enhances due to the formation of nanoaggregates, which is applicable to test the activity of the enzymes. In addition, the theranostic attributes of released AIEgens can be further exploited for imaging-guided therapy [56].

**Figure 3:**
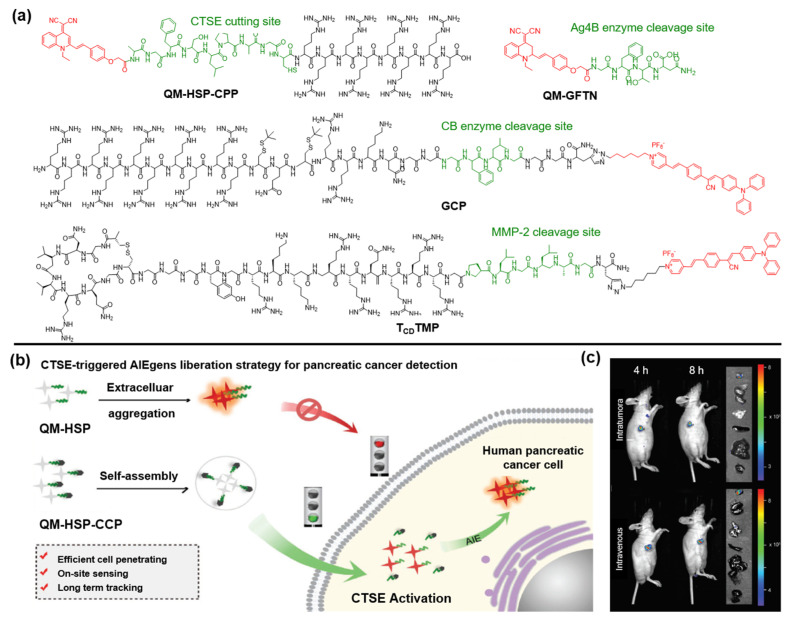
(**a**) Chemical structures of enzyme-activatable AIEgen-peptide bioconjugates. (**b**) CTSE-responsive bioconjugate (QM-HSP-CPP) for PC cancer detection. (**c**) Schematic diagram of fluorescence tracking endogenous CTSE after intra-tumoral or intravenous injection of QM-HSP-CPP. Copyright 2022, Wiley-VCH.

Zhu and coworkers designed an AIE-type activatable probe, QM-HSP-CPP, in which QM serves as an AIE core, HSP offers the specific recognition site of Cathepsin E (CTSE) that is overexpressed in pancreatic cancer (PC), and CPP guarantees the desirable amphiphilic characteristic for ensuring cell/tissue penetrating ability (Figure 3a) [57]. After incubation with SW1990 cells for different durations, probe QM-HSP-CPP was gradually cleaved by CTSE accompanied by the release of hydrophobic QM-HSP. The accumulation of QM-HSP in the physiological environment leads to the boosting of AIE signals with the prolongation of the accumulation time (Figure 3b). On the contrary, the mere usage of hydrophobic QM-HSP did not even guarantee the uptake efficiency by cells after incubation for 24 h. Subsequently, owing to the overexpression of endogenous CTSE in tumor sites, fluorescence signals were distinctly observed in the tumor site upon intravenous or intra-tumoral administration of QM-HSP-CPP for 4 h and remained at almost the same intensities within 8 h. By analysis of the fluorescence images of the tumor and normal organs, it was confirmed that the fluorescent signal was chiefly located in the tumor tissue (Figure 3c). Overall, the authors rationally designed an AIE probe for long-term monitoring of endogenous CTSE enzymes in human PC cells.

Recently, Wang et al. reported a strategy to monitor autophagy by synthesizing the Atg4B-responsive AIE probe QM-GFTN [58]. The incorporated GFTN peptide endows the probe with good hydrophilicity. Upon specific cleavage by Atg4B, the aggregation of the QM unit emitted intensive fluorescence due to its intrinsic AIE nature. In this regard, owing to the high specific response for Atg4B, it performed well in effectively distinguishing “autophagy active” states from “autophagy inactive” states.

Moreover, Hu and coworkers reported a multiple mechanism-based strategy by preparing a caged AIE-peptide probe (GCP) which can self-assemble with miR-140 to form GCP/miR-140 NPs [59]. Cleaved by CB enzymes, the structure of GCP/miR-140 dissolved with the release of caged GO203 peptide and miR-140. Further experiments demonstrated that the miR-140 downregulated the PD-L1 expression by suppressing its translation process. Meanwhile, PyTPA-mediated photodynamic therapy (PDT) could effectively activate the immune system to achieve strengthened immunotherapy. Similarly, Dai et al. also proposed a therapeutic protocol by designing a modular peptide probe (TCDTMP) which can be self-assembled into NPs after loading in miR-145-5p or VEGF siRNA [60].

### 3.2. Targetable AIEgen-Peptide Bioconjugates

Targetable identification of specific biomarkers can dramatically improve drug delivery efficiency and can be used as an efficient drug delivery method [61]. Targetable AIE-peptide bioconjugates usually contain two parts: (1) targeting modules to recognize specific cellular or biomarkers and (2) AIE-active fluorophores [62].

**Figure 4:**
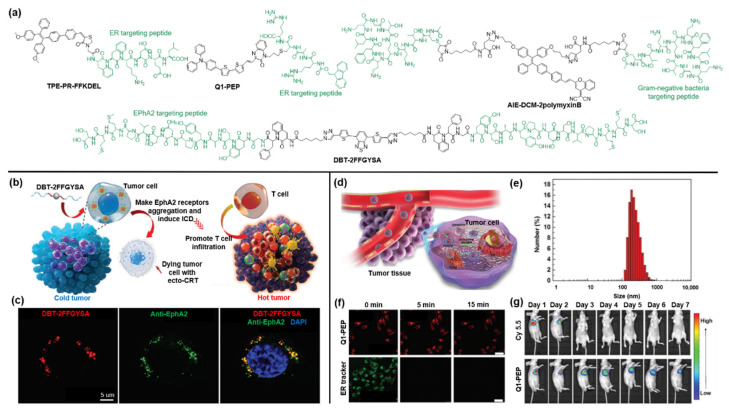
(**a**) Chemical structure of targetable AIEgen-peptide bioconjugates. (**b**) Schematic illustration of the working mechanism of DBT-2FFGYSA. (**c**) CLSM images of PC-3 cancer cells incubated with DBT-2FFGYSA. Copyright 2021, Wiley-VCH. (**d**) Schematic diagram of the working mechanism of Q1-PEP. (**e**) Particle size of Q1-PEP NPs in deionized water. (**f**) Photostability of Q1-PEP nanodots compared to ER tracker. (**g**) Photostability of Q1-PEP nanodots in contrast to Cy 5.5 in vivo. Copyright 2018, Wiley-VCH.

In most cancer cells, the tyrosine kinase Eph receptor A2 (EphA2) is overexpressed and plays a crucial part in deteriorating into cancer malignancy. Ding and coworkers designed a self-assembled peptide anticancer agent, named DBT-2FFGYSA, in which 4,7-di(thiophene-2-yl)-2,1,3-benzothiadiazole (DBT) functions as the central fluorophore incorporated with two peripheral peptides (FFGYSA) for specifically targeting EphA2 receptors (Figure 4a) [63]. Then, due to the excellent self-assembly ability of DBT-2FFGYSA and cross-linking of the two peptide chains, the overexpressed EphA2 receptors can automatically form giant clusters from nanometers to micrometers, resulting in the signaling of the antitumor pathway. As shown in Figure 4b, the agent DBT-2FFGYSA could effectively convert immunocompromised tumors into hot tumors by inducing immunogenic cell death (ICD) of EphA2 receptor-overexpressing cancer cells and by recruiting large numbers of tumor-infiltrating T cells. Cell imaging was conducted by CLSM imaging after incubation with human prostate PC-3 cancer cells that were first stained with anti-EPhA2 monoclonal antibody and fluorescent secondary antibody. As depicted in Figure 4c, it was found that intracellular DBT fluorescence (red pseudo-color) was in good accordance with the fluorescence of the antibody (green pseudo-color).

As an important organelle, the endoplasmic reticulum (ER) is involved in many biological processes. Studies have revealed that oxidative stress in the specific ER of cancer cells can enhance the ICD effect of cancer immunotherapy [64]. Ding et al. synthesized an ER-targeted AIE probe as an efficient ICD inducer for tumor immunotherapy by coupling an ER-targeting peptide (FFKDEL) with an AIE photosensitizer (TPE-PR-COOH) [65]. Besides, Zhang and coworkers developed nanodot Q1-PEP with ER-targeting capability by conjugating quinoxalinone scaffold with a Fmoc-protected oligopeptide to observe intracellular vesicular transport [66]. As shown in Figure 4d,e, the as-prepared nanodots showed a size of around 100 nm and were first taken up by cells, then escaped from the endosome owing to the proton sponge effect, and finally entered the ER, which can be exploited to track the vesicular transportation [67]. To evaluate the potential of nanoparticles as a visualization agent, researchers compared the photostability of nanodots and the commercial ER tracker under continuous light exposure for 15 min in MCF-7 cells (Figure 4f). The results suggested that the fluorescence signal of the Q1-PEP probe was barely attenuated during the irradiation, while the fluorescence of the commercial ER tracker was severely bleached. After that, an in vivo long-term tracking experiment was conducted. As shown in Figure 4g, the accumulation of NIR fluorescence of Q1-PEP nanodots can be clearly observed at the tumor site, and an evident fluorescence signal can still be detected even after 7 days. In contrast, the fluorescence signal of commercial dye Cy5.5 rapidly disappeared at 3 days post-injection. These results further confirmed the superior stability of Q1-PEP against photobleaching.

Due to the abuse of antibiotics, the resistance to antibiotics has posed a serious threat to antimicrobial therapy. The advent of bacterial identification can effectively overcome this challenge by identifying the source of the infection. Ding et al. designed and synthesized a peptide-based AIE bioprobe (AIE-DCM-2polymyxinB) by coupling AIEgen AIE-DCM with negative bacterium-targeting peptides of polymyxinB [68]. The strong specific binding of polymyxin B to lipopolysaccharide (LPS) confers it the ability to selectively target Gram-negative bacteria. Subsequently, the AIEgens can produce intensive fluorescence due to the RIM effect under the strong interaction between the probe and Gram-negative bacteria. Meanwhile, the probe AIE-DCM-2polymyxinB exhibited an excellent photodynamic anti-Gram-negative bacteria effect by generating efficient ROS.

## 4. AIEgen-Protein Bioconjugates

**Figure 5:**
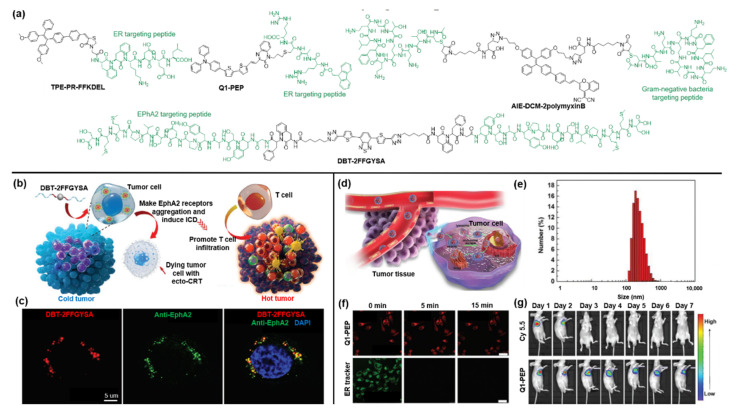
(**a**) Chemical structures of AIEgens reported recently to conjugate with proteins. (**b**) Absorption and emission spectra of mAb-CSPP bioconjugate. (**c**) Absorption and emission spectra of mAb-Cy3 bioconjugate. (**d**) Bright-field and fluorescent images of HCC827 cancer cells under different treatments. Copyright 2017, Royal Society of Chemistry. (**e**) Structures of AIE_810_NP-chicken IgY/AIE_810_NP-SARS-CoV-2 antigen and the diagram of lateral flow immunoassay for the detection of IgM and IgG. (**f**) Interpretation of different test results. Notably, the invalid test strip herein demonstrated only represents one case of a test with the result ‘invalid’. (**g**) The value of I_M_/I_C_ for the detection of IgM from 142 pre-COVID samples. (**h**) The value of I_G_/I_C_ for the detection of IgG from 142 pre-COVID samples. (**i**) Sensitivity of IgM/IgG testing for 172 serum samples from COVID-infected patients. Copyright 2021, American Chemical Society.

As one of the most important biomacromolecules in the living systems [69], proteins play an important role in the construction of the cell structure and substance and messages delivery and can serve as biosensors by modification with functional materials [70]. Benefiting from the advantages of NIR AIEgens, the bioconjugation between NIR AIEgens and specific proteins is considered as an appropriate method to manufacture smart biosensors, and numerous NIR AIEgen-protein based biosensors have been reported over the past few years (Figure 5a) [71,72,73,74,75].

The development of bioimaging with high SNR is urgently needed and remains challenging [76]. The bioconjugation between the monoclonal antibody (mAb) and the bioimaging probe is considered as an admirable strategy to design a molecular imaging probe with high SNR benefiting from the outstanding targeting specificity of antibodies. Recently, a water-soluble mAb-NIR AIEgen conjugate (mAb-CSPP) with “turn on” and “wash-free” characteristics was designed for specific cancer imaging [71]. In this work, mAb-CSPP was prepared by the conjugation between NHS-functionalized CSPP and cetuximab, and mAb-Cy3 with the “always-on” characteristic was also formulated as a control. The results of SDS-PAGE revealed that the cetuximabs were successfully conjugated with CSPP/Cy3. As illustrated in Figure 5b,c, compared with mAb-Cy3, mAb-CSPP possessed larger Stokes shift and a bathochromic shifted emission spectrum, which significantly reduced self-absorption and the cell autofluorescence. The water-soluble mAb-CSPP is non-emissive in aqueous solution, but it emitted energetically after the internalization induced by epidermal growth factor receptor (EGFR, overexpression in tumor cancer cells)-mediated endocytosis, which endows mAb-CSPP with the “turn on” characteristic. Subsequently, mAb-CSPP and mAb-Cy3 were utilized for HCC827 cancer cell imaging (Figure 5d). As expected, mAb-Cy3 with the “always on” characteristic exhibited a high background fluorescence signal with or without washing of PBS, and it was hard to capture the clear signals of cancer cells. In sharp contrast, neglectable background fluorescence of mAb-CSPP was observed, and signals were only detected in HCC827 cells with a high SNR due to the overexpressed EGFR, indicating the superiority of the “turn on” characteristic. The CLSM imaging also revealed that mAb-CSPP was mainly located in lysosome after incubation for 4 h and would migrate into the mitochondria at 24 h after hydrolysis in the lysosome. This work provided a new strategy to prepare bioimaging probes for specific cancer cells with the “turn on” characteristic and demonstrated favorable imaging performance with a high SNR.

The levels of IgM and IgG can serve as important indicators for infection in the early stage, and the detection of IgM/IgG is considered as an admirable and alternative method to diagnose COVID-19 [77,78]. Recently, a lateral flow immunoassay mediated by an NIR AIEgen-antigen probe for early detection of IgM/IgG was reported by Chen and coworkers [72]. The fluorescence probes AIE_810_NP-chicken and IgY/AIE_810_NP-SARS-CoV-2 antigen were facilely synthesized and employed to carry out the NIR lateral flow immunoassay, which could effectively eliminate the autofluorescence from the nitrocellulose membrane and the biosample. As illustrated in Figure 5e, the IgM/IgG would be initially captured by AIE_810_NP-SARS-CoV-2 antigen, after which it would be trapped by the mouse anti-human IgM/mouse anti-human IgG immobilized on the M/G line to indicate the IgM/IgG positivity (Figure 5f). The AIE_810_NP-chicken IgY would bind to goat anti-chicken IgY (immobilized on the C line) specifically to act as a control signal. After the optimization of immunoreaction conditions, further analysis of IgM/IgG was subsequently implemented with the assistance of the AIE_810_NP-based test strip for 142 pre-COVID serum samples. The experimental results revealed that the threshold for the detection of IgM and IgG is 0.200 (I_M_/I_C_) and 0.737 (I_G_/I_C_) (Figure 5g,h, I_M_, I_G_, and I_C_ are the fluorescence intensities of the M line, G line, and C line, respectively), and the limit of detection of IgM/IgG is 0.236 and 0.125 μg mL^−1^, respectively. Furthermore, 172 serum samples from patients infected with SARS-CoV-2 were tested by the AIE_810_NP-based test strip, ELISA, and AuNP-based test strips (Figure 5i). The sensitivity of the AIE_810_NP-based test strip is 78% and 95% in terms of the detection of IgM and IgG, respectively, which is comparable with ELISA (85% and 95%) and much better than AuNP-based test strips (41% and 85%). This work provided an alternative way to diagnose COVID-19 with considerable sensitivity based on the AIEgen-protein bioconjugate.

## 5. AIEgen-DNA Bioconjugates

DNA is another essential biological macromolecule in living systems, which carries the genetic information for the synthesis of RNA and proteins. Interestingly, over the past few years, DNA has emerged as an ideal candidate to construct functional materials through bioconjugation [79,80]. Nowadays, the bioconjugation between DNA and powerful AIEgen has invoked widespread research interests, and some prominent AIEgen-DNA bioconjugates have been developed.

**Figure 6:**
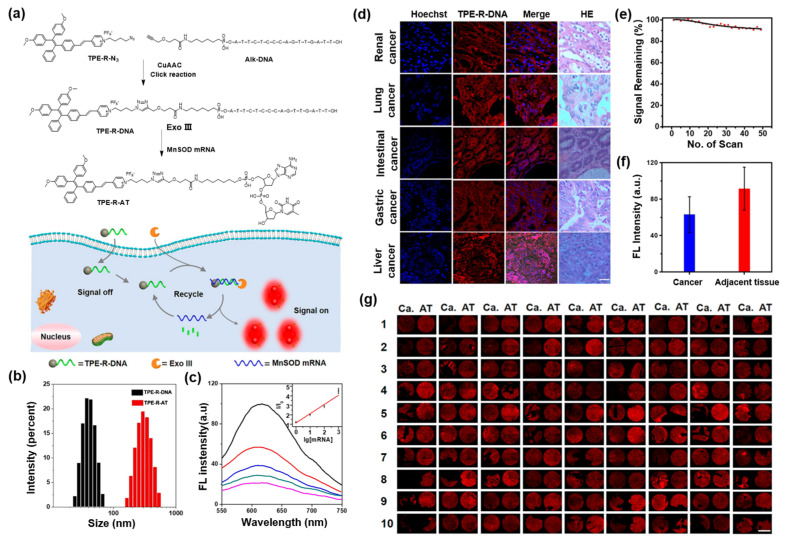
(**a**) Synthetic route of TPE-R-DNA, TPE-R-AT, and the diagram of detecting MnSOD mRNA. (**b**) Size distributions of TPE-R-DNA (left) and TPE-R-AT (right). (**c**) Photoluminescence spectra of TPE-R-DNA in the presence of Exo III and different concentrations of MnSOD mRNA (0−1000 pM). (**d**) CLSM images of many cancer tissues and their HE staining images. (**e**) Fluorescent signal remaining percent of TPE-R-DNA in liver cancer tissue with increasing scanning numbers. (**f**) Fluorescence intensity of cancer tissues and their adjacent tissues. (**g**) CLSM images of renal cancer tissues (Ca) and their adjacent tissues (AT). Copyright, 2018, American Chemical Society.

MnSOD is one of the key antioxidant enzymes involved in the conversion of superoxide radicals to keep cells from the destruction of ROS. MnSOD mRNA is in charge of the transcription of MnSOD, and therefore the MnSOD mRNA expression level is considered as an important index for cancer diagnosis [81,82]. Recently, a NIR AIEgen-DNA conjugate (TPE-R-DNA) was reported for cancer tissue imaging and prognosis analysis by detecting the mRNA expression level in tissues [83]. As displayed in Figure 6a, the amphiphilic TPE-R-DNA was first synthesized via the copper-catalyzed azide-alkyne click reaction between TPE-R-N_3_ and Alk-DNA, where the hydrophilic Alk-DNA with a complementary base sequence of MnSOD mRNA acted as the recognition portion, and the TPE moiety would endow the conjugate with AIE properties. In the presence of MnSOD mRNA and exonuclease III (Exo III) simultaneously, the amphiphilic TPE-R-DNA undergoes a hydrolysis reaction to form the hydrophobic TPE-R-AT, which was demonstrated by dynamic light scattering measurements (Figure 6b) and led to “turn on” of fluorescence emission. Notably, the experimental results exhibited a good linear correlation between fluorescence intensity and the concentration of mRNA, ranging from 0 to 1000 pM, with the detection limit lowered to 0.6 pM (Figure 6c). As shown in Figure 6d,e, TPE-R-DNA exhibited admirable photostability during 48 scans, and obvious red fluorescence signals were observed from all the cancer tissues incubated with the TPE-R-DNA, indicating the universality of the TPE-R-DNA probe for mRNA detection in tissue. Furthermore, the mRNA expression levels of both cancer tissue samples and adjacent normal tissue samples from patients were analyzed. As illustrated in Figure 6f,g, the fluorescence intensity of adjacent normal tissue was higher than that of renal cancer tissue because of the higher mRNA expression in adjacent normal tissues, which is consistent with previous reports. Undoubtedly, this work offered a platform for mRNA detection based on the AIEgen-DNA conjugate, exhibiting great potential for cancer tissue imaging and prognosis of gene-related diseases.

**Figure 7:**
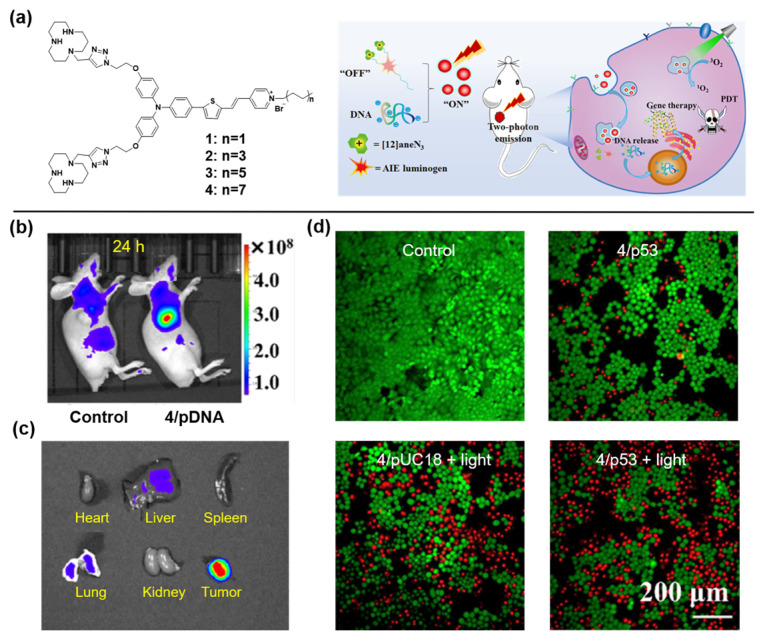
(**a**) Chemical structures of vectors 1–4. (**b**) In vivo imaging of tumor-bearing mice at 24 h post-injection of vector 4/DNA NPs. (**c**) Ex vivo biodistribution of various organs and tumor tissue from tumor-bearing mice at 24 h post-injection with vector 4/DNA NPs. (**d**) HeLa cells stained by calcein-AM (green, live) and PI (red, dead). Copyright, 2021, American Chemical Society.

Gene therapy is an emerging and powerful therapeutic strategy [84]. However, gene vectors with high efficiency and admirable compatibility are still urgently needed for the clinical applications [85]. A series of NIR AIEgens were synthesized to serve as fluorescent vectors by Tang et al. [86]. As illustrated in Figure 7a, the loose nucleic acid would be condensed into positively charged nanoparticles in the presence of aneN_3_ [12], and then internalized into the cell. Owing to the electrostatic interaction between the negatively charged endosomal membrane and the positively charged vector/DNA complexes, the genetic cargo and AIEgens would be released. Benefiting from the best photophysical properties, vector 4 was selected as the representative to carry out further studies. Surprisingly, the gene transfection efficiency of vector 4 was 6.7 times that of the commercial transfection agent Lipofectamine 2000. At 24 h post-injection with vector 4/DNA for the tumor-bearing mice, an obvious fluorescence signal was observed at the tumor site under light irradiation (Figure 7b), and ex vivo fluorescence imaging revealed that only tumor tissue intensely emitted (Figure 7c). Furthermore, the vector 4/DNA complexes also showed admirable combined PDT and gene therapy for HeLa cells, which was confirmed by the standard MTT assay (Figure 7d).

## 6. Other Systems

**Figure 8:**
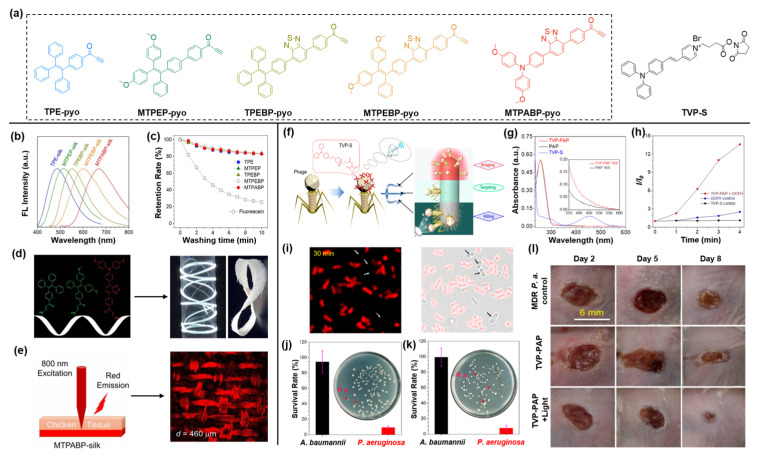
(**a**) Chemical structures of AIE-Pyo series and TVP-S. (**b**) Normalized fluorescence spectra of AIEgen-silks. (**c**) Fluorescence retention proportion of dyes after washing with soapy water. (**d**) Preparation and photos of WLE silk. (**e**) Two-photon fluorescent images of MTPABP-silk through the chicken tissues of 460 μm. Copyright 2021, Wiley-VCH. (**f**) Diagram of bacterial imaging, targeting, and killing driven by TVP-PAP. (**g**) Absorption spectra of TVP-PAP, PAP, and TVP-S. (**h**) DCFH for ROS detection of TVP-PAP. (**i**) Fluorescence imaging of *P. aeruginosa* and *A. baumanni* co-incubated with TVP-PAP for 30 min. (**j**) The survival rate and colonies of *P. aeruginosa* and *A. baumanni* co-incubated with TVP-PAP in the present light irradiation for 30 min. (**k**) The survival rates and colonies of *P. aeruginosa* and *S. aureus* co-incubated with TVP-PAP in the present light irradiation for 30 min. (**l**) The wound-healing rates at days 2, 5, and 8 under different treatments. Copyright, 2020, American Chemical Society.

Silkworm silk has been used in textile applications for a long time because of its unique mechanical strength, exceedingly good biocompatibility, optical transparency, and controllable biodegradability [87]. The functionalization of silk is a promising strategy to construct advanced material, especially fluorescent silks with great potential in functional bio-optical devices [88,89]. Recently, a series of fluorescent silks with full-color emission were prepared by metal-free bioconjugation [90]. As shown in Figure 8a (left), five AIEgens with activated alkyne groups were synthesized, and the emission wavelengths of them covered the whole visible region. The widespread amine groups on the surface of silk proteins can efficiently react with activated alkynes in a facile manner. As excepted, after soaking with AIEgens solutions at room temperature overnight, fluorescent silks (AIEgen-silks) were obtained through the metal-free bioconjugation between activated alkynes and amine groups with intensive fluorescence covering the entire visible light region (Figure 8b). As illustrated in Figure 8c, in comparison with the fluorescein-silk obtained by hydrogen bonding, AIEgen-silks fabricated by covalent bonding showed much higher stability after 10 min of washing with soapy water, demonstrating the unique advantage of chemically conjugated AIEgen-silks. As is known, the attractive white light-emitting (WLE) materials can be constructed by mixing red, green, and blue emitters [91]. As depicted in Figure 8d, controlling the AIEgens with blue, green, and red emission at a molar ratio of 88:6:6, WLE silks with outstanding flexibility were manufactured successfully through metal-free bioconjugation. The two-photon fluorescence (2PF) imaging experiment of MTPABP-silk (Figure 8e) revealed that the structure of the silk was distinctly visualized through the chicken tissues of 460 μm, and the red fluorescence signal can be observed even at a thickness of 1200 μm. This work proposed a new method to facilely fabricate fluorescence silk through metal-free bioconjugation, but also demonstrated their great potential in fabricating WLE material and deep-tissue imaging.

Historically, humankind arduously battled deadly microbes for a long time until the discovery and widespread use of penicillin. However, it has been repeatedly verified that both harmful and beneficial microbes would be simultaneously eliminated by antibiotics. Moreover, the emergence of multidrug-resistant bacteria caused by the abuse and misuse of antibiotics has triggered a serious threat to humans [92,93,94]. Therefore, a new antibacterial agent with high killing efficacy for certain species of bacteria is urgently needed. Tang et al., prepared a novel antimicrobial drug by the bioconjugation of AIEgens and phage, which can realize the specific imaging and killing of *P. aeruginosa* [95]. As one kind of virus, phage can target its hosts (including bacteria, fungi, algae, and others) with superb specificity and then attack them [96]. In view of the superiorities of AIEgens in microbial detection and therapy demonstrated by substantial studies, the AIEgen-phage bioconjugate is considered as a win–win integration [97]. As shown in Figure 8a,f, the activated carboxyl group in TVP-S would react with the amino group widely distributed in the protein shell of phage to prepare the TVP−PAP probe, which was further confirmed by the absorption spectrum analysis. The TVP-PAP possessed robust ROS generation efficiency (Figure 8h), which is inherited from TVP-S. As demonstrated in Figure 8i, after incubation with TVP-PAP for 30 min, a red fluorescence signal was distinctly detected from *P. aeruginosa*, but no fluorescence signal was observed from *A. baumannii*. Furthermore, in the presence of light illumination for 30 min, almost all the *P. aeruginosa* was killed, while few *A. baumannii* and *S. aureus* were eliminated (Figure 8j,k), demonstrating the outstanding targeted killing ability of TVP-PAP. Considering the remarkable bacteria targeting and elimination ability, TVP-PAP was selected to implement an in vivo antibacterial assay for *P. aeruginosa* and *MDR P. aeruginosa*-infected animal models. As shown in Figure 8l, at day 8 post-injection of TVP-PAP, the wound-healing rates for both *MDR P. aeruginosa* and *P. aeruginosa* infection were more than 90%. Certainly, this novel AIEgen-phage conjugated strategy discussed here will offer a universal approach for producing advanced antibacterial agents.

## 7. Summary and Perspective

AIE bioconjugates have grown into promising candidates for biomedical applications owing to the integrated advantages of AIEgens and biomolecules. In this review, we summarized several types of AIE bioconjugates, focusing on those emitting NIR fluorescence and highlighting their applications, especially in biomedicine.

While remarkable progresses have been made, there are still many challenges and limitations. For example, how can we facilely formulate a modulable synthesis route to obtain AIEgens with desired properties? How do we promote the bioconjugation in a biocompatible fashion (e.g., additive-free and smoothly proceeding, especially in the physiological environment)? In future research, considerable attempts should be devoted to the following aspects: (1) On the basis of the sophisticated functions of AIE bioconjugates, we encourage more efforts to enrich the categories of AIE bioconjugates. AIEgens possessing luminescent characteristics such as ultra-high brightness, high tissue penetration ability, two-photon or three-photon imaging capacity, and biological species with specific functionality are attractive. (2) Modulable synthesis of AIEgens with various functional groups such as amino group, carboxyl group, and azide group will undoubtedly create new possibilities in the quest for affording useful AIE bioconjugates. (3) Many facile ligation approaches based on the feature of biological species need to be developed. Taking the amino-yne reaction as an example, this click-type reaction allows for the conjugation process to proceed smoothly. (4) Bioconjugation spontaneously processed in live cells may confer the diagnosis of disease in a highly efficient way. With these prospects, it is anticipated that this review will prompt more innovative thoughts to further advance this area of research.

## References

[1] Giepmans B.N.G., Adams S.R., Ellisman M.H., Tsien R.Y. (2006). The Fluorescent Toolbox for Assessing Protein Location and Function. Science.

[2] Lee M.H., Kim J.S., Sessler J.L. (2015). Small molecule-based ratiometric fluorescence probes for cations, anions, and biomolecules. Chem. Soc. Rev..

[3] Etzioni R., Urban N., Ramsey S., McIntosh M., Schwartz S., Reid B., Radich J., Anderson G., Hartwell L. (2003). The case for early detection. Nat. Rev. Cancer.

[4] Beatriz S.P., Luis N., Leonor C., Laura M., Elena M., Yolanda F.N. (2016). Imaging Techniques and Scanning Electron Microscopy as Tools for Characterizing a Si-Based Material Used in Air Monitoring Applications. Materials.

[5] Garg P.K., Singh S.K., Prakash G., Jakhetiya A., Pandey D. (2016). Role of positron emission tomography-computed tomography in non-small cell lung cancer. World J. Methodol..

[6] Kolokythas O., Gauthier T., Fernandez A.T., Xie H., Timm B.A., Cuevas C., Dighe M.K., Mitsumori L.M., Bruce M.F., Herzka D.A. (2008). Ultrasound-Based Elastography: A Novel Approach to Assess Radio Frequency Ablation of Liver Masses Performed With Expandable Ablation Probes. J. Ultrasound Med..

[7] Lusic H., Grinstaff M.W. (2013). X-ray-Computed Tomography Contrast Agents. Chem. Rev..

[8] Kobayashi H., Ogawa M., Alford R., Choyke P.L., Urano Y. (2010). New Strategies for Fluorescent Probe Design in Medical Diagnostic Imaging. Chem. Rev..

[9] Cormode D.P., Jarzyna P.A., Mulder W.J.M., Fayad Z.A. (2010). Modified natural nanoparticles as contrast agents for medical imaging. Adv. Drug Deliv. Rev..

[10] Wiederschain G.Y. (2011). The Molecular Probes handbook. A guide to fluorescent probes and labeling technologies. Biochemistry.

[11] Tsien R.Y. (2009). Constructing and Exploiting the Fluorescent Protein Paintbox (Nobel Lecture). Angew. Chem. Int. Ed..

[12] He L., Yang X., Xu K., Kong X., Lin W. (2017). A multi-signal fluorescent probe for simultaneously distinguishing and sequentially sensing cysteine/homocysteine, glutathione, and hydrogen sulfide in living cells. Chem. Sci..

[13] Chinen A.B., Guan C.M., Ferrer J.R., Barnaby S.N., Merkel T.J., Mirkin C.A. (2015). Nanoparticle Probes for the Detection of Cancer Biomarkers, Cells, and Tissues by Fluorescence. Chem. Rev..

[14] Gnach A., Lipinski T., Bednarkiewicz A., Rybka J., Capobianco J.A. (2015). Upconverting nanoparticles: Assessing the toxicity. Chem. Soc. Rev..

[15] Xu W., Wang D., Tang B.Z. (2021). NIR-II AIEgens: A Win-Win Integration towards Bioapplications. Angew. Chem. Int. Ed..

[16] Yan D., Wang M., Wu Q., Niu N., Li M., Song R., Rao J., Kang M., Zhang Z., Zhou F. (2022). Multimodal Imaging-Guided Photothermal Immunotherapy Based on a Versatile NIR-II Aggregation-Induced Emission Luminogen. Angew. Chem..

[17] Wang D., Lee M.M.S., Shan G., Kwok R.T.K., Lam J.W.Y., Su H., Cai Y., Tang B.Z. (2018). Highly Efficient Photosensitizers with Far-Red/Near-Infrared Aggregation-Induced Emission for In Vitro and In Vivo Cancer Theranostics. Adv. Mater..

[18] Yan D., Xie W., Zhang J., Wang L., Wang D., Tang B.Z. (2021). Donor/π-Bridge Manipulation for Constructing a Stable NIR-II Aggregation-Induced Emission Luminogen with Balanced Phototheranostic Performance. Angew. Chem. Int. Ed..

[19] Mei J., Huang Y., Tian H. (2018). Progress and Trends in AIE-Based Bioprobes: A Brief Overview. ACS Appl. Mater. Interfaces.

[20] Luo J., Xie Z., Lam J.W.Y., Cheng L., Chen H., Qiu C., Kwok H.S., Zhan X., Liu Y., Zhu D. (2001). Aggregation-induced emission of 1-methyl-1,2,3,4,5-pentaphenylsilole. Chem. Commun..

[21] Mei J., Leung N.L.C., Kwok R.T.K., Lam J.W.Y., Tang B.Z. (2015). Aggregation-Induced Emission: Together We Shine, United We Soar!. Chem. Rev..

[22] Chen J., Law C.C.W., Lam J.W.Y., Dong Y., Lo S.M.F., Williams I.D., Zhu D., Tang B.Z. (2003). Synthesis, Light Emission, Nanoaggregation, and Restricted Intramolecular Rotation of 1,1-Substituted 2,3,4,5-Tetraphenylsiloles. Chem. Mater..

[23] Leung N.L.C., Xie N., Yuan W., Liu Y., Wu Q., Peng Q., Miao Q., Lam J.W.Y., Tang B.Z. (2014). Restriction of Intramolecular Motions: The General Mechanism behind Aggregation-Induced Emission. Chem. Eur. J..

[24] Mei J., Hong Y., Lam J.W.Y., Qin A., Tang Y., Tang B.Z. (2014). Aggregation-Induced Emission: The Whole Is More Brilliant than the Parts. Adv. Mater..

[25] Wang D., Tang B.Z. (2019). Aggregation-Induced Emission Luminogens for Activity-Based Sensing. Acc. Chem. Res..

[26] Kwok R.T.K., Leung C.W.T., Lam J.W.Y., Tang B.Z. (2015). Biosensing by luminogens with aggregation-induced emission characteristics. Chem. Soc. Rev..

[27] Wang D., Su H., Kwok R.T.K., Hu X., Zou H., Luo Q., Lee M.M.S., Xu W., Lam J.W.Y., Tang B.Z. (2018). Rational design of a water-soluble NIR AIEgen, and its application in ultrafast wash-free cellular imaging and photodynamic cancer cell ablation. Chem. Sci..

[28] Su X., Han T., Niu N., Li H., Wang D., Tang B.Z. (2021). Facile Multicomponent Polymerizations toward Multifunctional Heterochain Polymers with α,β-Unsaturated Amidines. Macromolecules.

[29] Wang Y., Yan D., Wang L., Wang D., Tang B.Z. (2021). Aggregation-Induced Emission Luminogens Sensitized Quasi-2D Hybrid Perovskites with Unique Photoluminescence and High Stability for Fabricating White Light-Emitting Diodes. Adv. Sci..

[30] Lee M.M.S., Yan D., Chau J.H.C., Park H., Ma C.C.H., Kwok R.T.K., Lam J.W.Y., Wang D., Tang B.Z. (2020). Highly efficient phototheranostics of macrophage-engulfed Gram-positive bacteria using a NIR luminogen with aggregation-induced emission characteristics. Biomaterials.

[31] Yan S., Sun P., Niu N., Zhang Z., Xu W., Zhao S., Wang L., Wang D., Tang B.Z. (2022). Surfactant-Inspired Coassembly Strategy to Integrate Aggregation-Induced Emission Photosensitizer with Organosilica Nanoparticles for Efficient Theranostics. Adv. Funct. Mater..

[32] Zhang M., Wang W.T., Mohammadniaei M., Zheng T., Zhang Q.C., Ashley J., Liu S.J., Sun Y., Tang B.Z. (2021). Upregulating Aggregation-Induced-Emission Nanoparticles with Blood–Tumor-Barrier Permeability for Precise Photothermal Eradication of Brain Tumors and Induction of Local Immune Responses. Adv. Mater..

[33] Liu Y., Deng C., Tang L., Qin A., Hu R., Sun J.Z., Tang B.Z. (2011). Specific Detection of d-Glucose by a Tetraphenylethene-Based Fluorescent Sensor. J. Am. Chem. Soc..

[34] Ou X., Hong F., Zhang Z., Cheng Y., Zhao Z., Gao P., Lou X., Xia F., Wang S. (2017). A highly sensitive and facile graphene oxide-based nucleic acid probe: Label-free detection of telomerase activity in cancer patient’s urine using AIEgens. Biosens. Bioelectron..

[35] Li Y., Kwok R.T.K., Tang B.Z., Liu B. (2013). Specific nucleic acid detection based on fluorescent light-up probe from fluorogens with aggregation-induced emission characteristics. RSC Adv..

[36] Gu X., Zhao E., Zhao T., Kang M., Gui C., Lam J.W.Y., Du S., Loy M.M.T., Tang B.Z. (2016). A Mitochondrion-Specific Photoactivatable Fluorescence Turn-On AIE-Based Bioprobe for Localization Super-Resolution Microscope. Adv. Mater..

[37] Shi H., Liu J., Geng J., Tang B.Z., Liu B. (2012). Specific Detection of Integrin αvβ3 by Light-Up Bioprobe with Aggregation-Induced Emission Characteristics. J. Am. Chem. Soc..

[38] Shi H., Kwok R.T.K., Liu J., Xing B., Tang B.Z., Liu B. (2012). Real-Time Monitoring of Cell Apoptosis and Drug Screening Using Fluorescent Light-Up Probe with Aggregation-Induced Emission Characteristics. J. Am. Chem. Soc..

[39] Kang M., Zhou C., Wu S., Yu B., Zhang Z., Song N., Lee M.M.S., Xu W., Xu F.-J., Wang D. (2019). Evaluation of Structure–Function Relationships of Aggregation-Induced Emission Luminogens for Simultaneous Dual Applications of Specific Discrimination and Efficient Photodynamic Killing of Gram-Positive Bacteria. J. Am. Chem. Soc..

[40] Huang J., He B., Zhang Z., Li Y., Kang M., Wang Y., Li K., Wang D., Tang B.Z. (2020). Aggregation-Induced Emission Luminogens Married to 2D Black Phosphorus Nanosheets for Highly Efficient Multimodal Theranostics. Adv. Mater..

[41] Liu H., Xiong L.H., Kwok R.T.K., He X., Lam J.W.Y., Tang B.Z. (2020). AIE Bioconjugates for Biomedical Applications. Adv. Opt. Mater..

[42] Aron A.T., Ramos-Torres K.M., Cotruvo J.A., Chang C.J. (2015). Recognition- and Reactivity-Based Fluorescent Probes for Studying Transition Metal Signaling in Living Systems. Acc. Chem. Res..

[43] Ding D., Li K., Liu B., Tang B.Z. (2013). Bioprobes Based on AIE Fluorogens. Acc. Chem. Res..

[44] He S., Sharpless N.E. (2017). Senescence in Health and Disease. Cell.

[45] Kamiya M., Asanuma D., Kuranaga E., Takeishi A., Sakabe M., Miura M., Nagano T., Urano Y. (2011). β-Galactosidase Fluorescence Probe with Improved Cellular Accumulation Based on a Spirocyclized Rhodol Scaffold. J. Am. Chem. Soc..

[46] Spergel D.J., Krüth U., Shimshek D.R., Sprengel R., Seeburg P.H. (2001). Using reporter genes to label selected neuronal populations in transgenic mice for gene promoter, anatomical, and physiological studies. Prog. Neurobiol..

[47] Yao Y., Zhang Y., Yan C., Zhu W.-H., Guo Z. (2021). Enzyme-activatable fluorescent probes for β-galactosidase: From design to biological applications. Chem. Sci..

[48] Gao Z., Gao H., Zheng D., Xu T., Chen Y., Liang C., Wang L., Ding D., Yang Z. (2020). β-galactosidase responsive AIE fluorogene for identification and removal of senescent cancer cells. Sci. China Ser. B Chem..

[49] Fu W., Yan C., Zhang Y., Ma Y., Guo Z., Zhu W.-H. (2019). Near-Infrared Aggregation-Induced Emission-Active Probe Enables in situ and Long-Term Tracking of Endogenous β-Galactosidase Activity. Front. Chem..

[50] Chan J., Dodani S.C., Chang C.J. (2012). Reaction-based small-molecule fluorescent probes for chemoselective bioimaging. Nat. Chem..

[51] Yang J., Wei J., Luo F., Dai J., Hu J.-J., Lou X., Xia F. (2020). Enzyme-Responsive Peptide-Based AIE Bioprobes. Top. Curr. Chem..

[52] Braun G.B., Sugahara K.N., Yu O.M., Kotamraju V.R., Mölder T., Lowy A.M., Ruoslahti E., Teesalu T. (2016). Urokinase-controlled tumor penetrating peptide. J. Control. Release.

[53] Wang F., Gómez-Sintes R., Boya P. (2018). Lysosomal membrane permeabilization and cell death. Traffic.

[54] Hengartner M.O. (2000). The biochemistry of apoptosis. Nature.

[55] Hadler-Olsen E., Winberg J.-O., Uhlin-Hansen L. (2013). Matrix metalloproteinases in cancer: Their value as diagnostic and prognostic markers and therapeutic targets. Tumor Biol..

[56] Shi H., Zhao N., Ding D., Liang J., Tang B.Z., Liu B. (2013). Fluorescent light-up probe with aggregation-induced emission characteristics for in vivo imaging of cell apoptosis. Org. Biomol. Chem..

[57] Zhu Z., Wang Q., Chen X., Wang Q., Yan C., Zhao X., Zhao W., Zhu W. (2022). An Enzyme-Activatable Aggregation-Induced-Emission Probe: Intraoperative Pathological Fluorescent Diagnosis of Pancreatic Cancer via Specific Cathepsin E. Adv. Mater..

[58] Lyu Y., Chen X., Wang Q., Li Q., Wang Q., Li X., Zhu Z., Yan C., Zhao X., Zhu W. (2022). Monitoring Autophagy with Atg4B Protease-Activated Aggregation-Induced Emission Probe. Adv. Funct. Mater..

[59] Dai J., Cheng Y., Wu J., Wang Q., Wang W., Yang J., Zhao Z., Lou X., Xia F., Wang S. (2020). Modular Peptide Probe for Pre/Intra/Postoperative Therapeutic to Reduce Recurrence in Ovarian Cancer. ACS Nano.

[60] Chen C., Ni X., Jia S., Liang Y., Wu X., Kong D., Ding D. (2019). Massively Evoking Immunogenic Cell Death by Focused Mitochondrial Oxidative Stress using an AIE Luminogen with a Twisted Molecular Structure. Adv. Mater..

[61] Xia F., Wu J., Wu X., Hu Q., Dai J., Lou X. (2019). Modular Design of Peptide- or DNA-Modified AIEgen Probes for Biosensing Applications. Acc. Chem. Res..

[62] Singh D.R., Kanvinde P., King C., Pasquale E.B., Hristova K. (2018). The EphA2 receptor is activated through induction of distinct, ligand-dependent oligomeric structures. Commun. Biol..

[63] Li J., Fang Y., Zhang Y., Wang H., Yang Z., Ding D. (2021). Supramolecular Self-Assembly-Facilitated Aggregation of Tumor-Specific Transmembrane Receptors for Signaling Activation and Converting Immunologically Cold to Hot Tumors. Adv. Mater..

[64] Kroemer G., Galluzzi L., Kepp O., Zitvogel L. (2013). Immunogenic Cell Death in Cancer Therapy. Annu. Rev. Immunol..

[65] Li J., Gao H., Liu R., Chen C., Zeng S., Liu Q., Ding D. (2020). Endoplasmic reticulum targeted AIE bioprobe as a highly efficient inducer of immunogenic cell death. Sci. China Ser. B Chem..

[66] Shi L., Gao X., Yuan W., Xu L., Deng H., Wu C., Yang J., Jin X., Zhang C., Zhu X. (2018). Endoplasmic Reticulum–Targeted Fluorescent Nanodot with Large Stokes Shift for Vesicular Transport Monitoring and Long-Term Bioimaging. Small.

[67] Copolovici D.M., Langel K., Eriste E., Langel Ü. (2014). Cell-Penetrating Peptides: Design, Synthesis, and Applications. ACS Nano.

[68] Bao P., Li C., Ou H., Ji S., Chen Y., Gao J., Yue X., Shen J., Ding D. (2021). A peptide-based aggregation-induced emission bioprobe for selective detection and photodynamic killing of Gram-negative bacteria. Biomater. Sci..

[69] Altschul S.F., Madden T.L., Schaffer A.A., Zhang J.H., Zhang Z., Miller W., Lipman D.J. (1997). Gapped BLAST and PSI-BLAST: A new generation of protein database search programs. Nucleic Acids Res..

[70] Zhang C., Vinogradova E.V., Spokoyny A.M., Buchwald S.L., Pentelute B.L. (2019). Arylation Chemistry for Bioconjugation. Angew. Chem. Int. Ed..

[71] Shi X.J., Yu C.Y.Y., Su H.F., Kwok R.T.K., Jiang M.J., He Z.K., Lam J.W.Y., Tang B.Z. (2017). A red-emissive antibody-AIEgen conjugate for turn-on and wash-free imaging of specific cancer cells. Chem. Sci..

[72] Chen R., Ren C.P., Liu M., Ge X.P., Qu M.S., Zhou X.B., Liang M.F., Liu Y., Li F.Y. (2021). Early Detection of SARS-CoV-2 Seroconversion in Humans with Aggregation-Induced Near-Infrared Emission Nanoparticle-Labeled Lateral Flow Immunoassay. ACS Nano.

[73] Wu W.B., Feng G.X., Xu S.D., Liu B. (2016). A Photostable Far-Red/Near-Infrared Conjugated Polymer Photosensitizer with Aggregation-Induced Emission for Image-Guided Cancer Cell Ablation. Macromolecules.

[74] Soleimaninejad H., Chen M.Z., Lou X.D., Smith T.A., Hong Y.N. (2017). Measuring macromolecular crowding in cells through fluorescence anisotropy imaging with an AIE fluorogene. Chem. Commun..

[75] Hu Q., Yao B.C., Owyong T.C., Prashanth S., Wang C.Y., Zhang X.Y., Wong W.W.H., Tang Y.H., Hong Y.N. (2021). Detection of Urinary Albumin Using a “Turn-on” Fluorescent Probe with Aggregation-Induced Emission Characteristics. Chem. Asian J..

[76] Kobayashi H., Longmire M.R., Ogawa M., Choyke P.L. (2011). Rational chemical design of the next generation of molecular imaging probes based on physics and biology: Mixing modalities, colors and signals. Chem. Soc. Rev..

[77] Reichert J.M., Dhimolea E. (2012). The future of antibodies as cancer drugs. Drug Discov. Today.

[78] Baselga J., Arteaga C.L. (2005). Critical update and emerging trends in epidermal growth factor receptor targeting in cancer. J. Clin. Oncol..

[79] Amanat F., Stadlbauer D., Strohmeier S., Nguyen T.H.O., Chromikova V., McMahon M., Jiang K.J., Arunkumar G.A., Jurczyszak D., Polanco J. (2020). A serological assay to detect SARS-CoV-2 seroconversion in humans. Nat. Med..

[80] Vashist S.K. (2020). In Vitro Diagnostic Assays for COVID-19: Recent Advances and Emerging Trends. Diagnostics.

[81] Lee H., Rho J., Messersmith P.B. (2009). Facile Conjugation of Biomolecules onto Surfaces via Mussel Adhesive Protein Inspired Coatings. Adv. Mater..

[82] Shull S., Heintz N.H., Periasamy M., Manohar M., Janssen Y.M.W., Marsh J.P., Mossman B.T. (1991). DIFFERENTIAL REGULATION OF ANTIOXIDANT ENZYMES IN RESPONSE TO OXIDANTS. J. Biol. Chem..

[83] Wang X.D., Dai J., Min X.H., Yu Z.H., Cheng Y., Huang K.X., Yang J.L., Yi X.Q., Lou X.D., Xia F. (2018). DNA-Conjugated Amphiphilic Aggregation-Induced Emission Probe for Cancer Tissue Imaging and Prognosis Analysis. Anal. Chem..

[84] Nayerossadat N., Maedeh T., Ali P.A. (2012). Viral and nonviral delivery systems for gene delivery. Adv. Biomed. Res..

[85] He D., Wagner E. (2015). Defined Polymeric Materials for Gene Delivery. Macromol. Biosci..

[86] Tang F., Liu J.-Y., Wu C.-Y., Liang Y.-X., Lu Z.-L., Ding A.-X., Xu M.-D. (2021). Two-Photon Near-Infrared AIE Luminogens as Multifunctional Gene Carriers for Cancer Theranostics. ACS Appl. Mater. Interfaces.

[87] Altman G.H., Diaz F., Jakuba C., Calabro T., Horan R.L., Chen J.S., Lu H., Richmond J., Kaplan D.L. (2003). Silk-based biomaterials. Biomaterials.

[88] Cheng L., Zhao H.P., Huang H.M., Li B., Li R.K.Y., Feng X.Q., Dai F.Y. (2019). Quantum dots-reinforced luminescent silkworm silk with superior mechanical properties and highly stable fluorescence. J. Mater. Sci..

[89] Amirikia M., Shariatzadeh S.M.A., Jorsaraei S.G.A., Mehranjani M.S. (2018). Auto-fluorescence of a silk fibroin-based scaffold and its interference with fluorophores in labeled cells. Eur. Biophys. J..

[90] Liu C.C., Bai H.T., He B.Z., He X.W., Zhang J.Y., Chen C., Qiu Y.P., Hu R., Zhao F.X., Zhang Y.X. (2021). Functionalization of Silk by AIEgens through Facile Bioconjugation: Full-Color Fluorescence and Long-Term Bioimaging. Angew. Chem. Int. Ed..

[91] Lin N.B., Hu F., Sun Y.L., Wu C.X., Xu H.Y., Liu X.Y. (2014). Construction of White-Light-Emitting Silk Protein Hybrid Films by Molecular Recognized Assembly among Hierarchical Structures. Adv. Funct. Mater..

[92] Kardas P., Devine S., Golembesky A., Roberts C. (2005). A systematic review and meta-analysis of misuse of antibiotic therapies in the community. Int. J. Antimicrob. Agents.

[93] Suez J., Zmora N., Zilberman-Schapira G., Mor U., Dori-Bachash M., Bashiardes S., Zur M., Regev-Lehavi D., Brik R.B., Federici S. (2018). Post-Antibiotic Gut Mucosal Microbiome Reconstitution Is Impaired by Probiotics and Improved by Autologous FMT. Cell.

[94] Yoshikawa T.T. (2002). Antimicrobial resistance and aging: Beginning of the end of the antibiotic era?. J. Am. Geriatr. Soc..

[95] He X.W., Yang Y.J., Guo Y.C., Lu S.G., Du Y., Li J.J., Zhang X.P., Leung N.L.C., Zhao Z., Niu G.L. (2020). Phage-Guided Targeting, Discriminative Imaging, and Synergistic Killing of Bacteria by AIE Bioconjugates. J. Am. Chem. Soc..

[96] Xiao P.H., Shen Z.P., Wang D.L., Pan Y.Z., Li Y., Gong J.Y., Wang L., Wang D., Tang B.Z. (2022). Precise Molecular Engineering of Type I Photosensitizers with Near-Infrared Aggregation-Induced Emission for Image-Guided Photodynamic Killing of Multidrug-Resistant Bacteria. Adv. Sci..

[97] Nobrega F.L., Vlot M., de Jonge P.A., Dreesens L.L., Beaumont H.J.E., Lavigne R., Dutilh B.E., Brouns S.J.J. (2018). Targeting mechanisms of tailed bacteriophages. Nat. Rev. Microbiol..

